# Initial activation of STAT2 induced by IAV infection is critical for innate antiviral immunity

**DOI:** 10.3389/fimmu.2022.960544

**Published:** 2022-09-05

**Authors:** Xinxin Li, Siya Liu, Kul Raj Rai, Wenzhuo Zhou, Song Wang, Xiaojuan Chi, Guijie Guo, Ji-Long Chen, Shasha Liu

**Affiliations:** ^1^ Fujian Agriculture and Forestry University, Fuzhou, China; ^2^ Key Laboratory of Animal Pathogen Infection and Immunology of Fujian Province, College of Animal Sciences, Fujian Agriculture and Forestry University, Fuzhou, China

**Keywords:** STAT2, influenza virus, kinase, MAPK12, innate immunity

## Abstract

STAT2 is an important transcription factor activated by interferons (IFNs) upon viral infection and plays a key role in antiviral responses. Interestingly, here we found that phosphorylation of STAT2 could be induced by several viruses at early infection stage, including influenza A virus (IAV), and such initial activation of STAT2 was independent of type I IFNs and JAK kinases. Furthermore, it was observed that the early activation of STAT2 during viral infection was mainly regulated by the RIG-I/MAVS-dependent pathway. Disruption of STAT2 phosphorylation at Tyr690 restrained antiviral response, as silencing STAT2 or blocking STAT2 Y690 phosphorylation suppressed the expression of several interferon-stimulated genes (ISGs), thereby facilitating viral replication. *In vitro* experiments using overexpression system or kinase inhibitors showed that several kinases including MAPK12 and Syk were involved in regulation of the early phosphorylation of STAT2 triggered by IAV infection. Moreover, when MAPK12 kinase was inhibited, expression of several ISGs was clearly decreased in cells infected with IAV at the early infection stage. Accordingly, inhibition of MAPK12 accelerated the replication of influenza virus in host. These results provide a better understanding of how initial activation of STAT2 and the early antiviral responses are induced by the viral infection.

## 1 Introduction

Influenza A virus (IAV) is widespread throughout the world and remains a major threat to human and animal health, causing significant annual mortality in severe cases (1, 2). Innate immunity is the front line of defense against viral infections. It uses evolutionarily highly conserved pattern recognition receptors (PRRs) to recognize specific components of bacteria or viruses to trigger immune responses (3). PRRs mainly include retinoic acid-induced gene type I receptors (RLRs), toll-like receptor (TLRs), nucleotide oligomeric domain (NOD)-like receptors (NLRs), C-type lectin receptors (CLRs), DNA-dependent activation of interferon regulatory factor 3 (DAI), and melanoma deficiency factor 2 (AIM2), etc (4–6). Activation of PRR-dependent pathway leads to the secretion of interferons (IFNs), inflammatory cytokines, and other mediators that recruit and activate effector cells, which contributes to pathogen elimination (7). In infected cells, panhandle structure of the IAV genome RNA can be detected by RIG-I (8). The genome of IAV can also be detected by other PRRs, such as TLR3 (9), TLR7 (10), and TLR8 (11). After binding to PRR, RIG-I can recruit mitochondrial antiviral signaling proteins (MAVS) by interacting with the CARD domain to initiate signaling pathways (12), thereby promoting the activation of multiple transcription factors, such as IRF3, IRF7, and NF-κB (13), which induce the production of cytokines and proinflammatory cytokines.

Signal transducer and activator of transcription 2 (STAT2) is a member of the STATs family, and like other members of the family, contains seven domains that are structurally and functionally conservative: N-terminal domain (NTD), coiled-helix domain (CCD), DNA-binding domain (DBD), crossover domain (LD), Src homologous 2 domain (SH2D), tyrosine phosphorylation site (pY), and transcriptional activation domain (TAD). STAT2, with a molecular weight of 113 kDa (14), has a variety of biological activities and is an important transcription factor implicated in the antiviral responses. Previous studies have shown that classical type I and type III IFNs bind to corresponding IFN receptors to activate Janus kinases (JAKs), including JAK1 and TYK2 (15, 16). Activated JAKs and STATs pathway mediates binding of the STAT1-STAT2 heterodimer with interferon regulator 9 (IRF9) to form the transcription factor complex ISGF3 (15, 17). ISGF3 binds to the IFN stimulating response element (ISRE) and controls the expression of numerous antiviral proteins (18), thereby establishing an antiviral state through a variety of mechanisms, including inhibition of viral transcription, translation and replication, and degradation of viral nucleic acids (19–21). In addition to its role in antiviral immunity, STAT2 has also been shown to be involved in the regulation of immune signaling. For example, STAT2 functions as a universal cytokine regulator by inhibiting STAT1 in multiple signaling pathways (22). Based on previous findings, Zika virus (ZIKV) NS5 protein induces the ubiquitination and degradation of STAT2, promotes STAT1 homodimer formation and enhances IFN-γ-induced ISG expression (23).

Although IFNs-activated JAK-STAT is a typical pathway for activation of innate immunity, many viruses have evolved strategies to evade the IFN signaling. For instance, paramyxovirus HPIV2 induces degradation of STAT2 protein (24). Similarly, V proteins from Hendra and Nipah viruses inactivate IFN signaling by directly inhibiting STAT1 and STAT2 functions (25, 26). The unique immune evasion mechanisms of these viruses suggest the importance of STAT2 for innate antiviral immunity. Previous reports have revealed that STAT2 is essential for TLR-induced inflammatory cytokines production in bone-marrow derived macrophage (BMDM) cell lines in a type I IFN receptor-independent manner, highlighting the prominent role of STAT2 independent of type I IFN (27). Moreover, there is evidence that some virus-induced ISGs are independent of IFN-mediated innate immune pathways (28, 29). Such as, the upregulation of ISG15 during HCMV infection occurred in an IFN-independent, IRF3-dependent manner (28, 30). These indicate that there exist IFN-independent mechanisms in response to viral infection. In addition, it is also thought that subcellular localization of MAVS determines the signaling activated during viral infection that controls ISG expression. For example, the expression of ISGs is mainly dependent on peroxisome-localized MAVS rather than mitochondria-localized MAVS during reovirus infection (31). Although extensive studies on STAT have been focused on the IFN-mediated JAKs over the past decades, a growing number of studies have suggested other pathways activating STAT. It is observed that cyclin-dependent kinase, as a positive regulator of IFN signaling, can directly affect STAT activation (32). SOCS1, an important suppressor of JAK-STAT signaling, is expressed earlier than cytokine secretion, implying that there may be a cytokine-independent pathway regulating STAT activity (33). Recently, it has also been shown that early activation of STAT1 during the viral infection is mediated by spleen tyrosine kinase (Syk) but not cytokine-activated JAK, providing new insights into the complex mechanisms underlying interaction between virus and host immune system (34).

In this study, we observed that virus-induced initial activation of STAT2 was independent of type I IFN signaling. Specifically, increased phosphorylation of STAT2 occurred at the early phase of infection with multiple viruses. Further experiments displayed that RIG-I/MAVS pathway regulated such activation of STAT2. In addition, we found that mitogen-activated protein kinase 12 (MAPK12) was involved in activating STAT2 and positively regulated the expression of some ISGs at the early phase of IAV infection. These results provide new evidence of how the early activation of STAT2-involved innate immunity is regulated during the viral infection.

## 2 Materials and methods

### 2.1 Cells

A549 (ATCC CCL-185), 293T (CRL -11268), MDCK (ATCC CRL -2935), and PK15 cells used in this study were all from American Type Culture Collection (ATCC). Cells were grown in monolayer culture in Dulbecco Modified Eagle Medium (DMEM) supplemented with 10% fetal bovine serum (FBS) (Gibco-BRL, Gaithersburg, MD, USA), 2 μg/mL trypsin, 100 U/mL penicillin and 100 g/mL streptomycin. Cells were cultured every two days, inoculated into porous plates one day before virus infection and further cultured at 37°C and 5% CO_2_.

### 2.2 Reagents

In this study, the following antibodies were mainly used: anti-flag (Protein Tech, Wuhan, 66008-3-IG), anti-STAT2 (Cell Signaling Technology, Danvers, MA, USA, 72604), anti-pSTAT2 (Cell Signaling Technology, Danvers, MA, USA, 88410), and anti-β-actin (TransGen Biotech, Beijing, HC201). The antibodies against NP protein of IAV and GE of PRV were produced by our laboratory. IFN-β was purchased from Sino Biologicals (Beijing), SB203580, INCB-018424, and CP690550 were from Selleck (Houston, TX, USA).

### 2.3 Virus amplification and Infection

IAV strains including A/WSN/33 (H1N1) (WSN), A/PR/8/34 (H1N1) (PR8), A/CA/04/09 (H1N1) (CA04), and H9N2 were cultured for 2 days in allantoic fluid of chicken embryo aged 9-10 days without specific pathogens. Pseudorabies virus (PRV) was propagated in MDCK cells and Muscovy Duck Reovirus (MDRV) was propagated in 293T cells, as previously described (35). For viral infection, cells were infected with virus at the indicated multiplicity of infection (MOI), mixed, 37°C adsorbed for 1 h, gently shaken every 15 min. Appropriate new maintenance solution was added to continue culture in 37°C and 5% CO_2_.

### 2.4 RNA interference and construction of stable cell lines

Lentiviral vector expressing specific shRNAs in pSIH-H1-GFP vector along with lentiviral packaging plasmids were transfected into 293T cells. The virus-containing supernatant was collected 48 h after transfection and infected A549 cells by centrifugation at 2200 rpm for 2 h, as previously described (36). The sequences used in the shRNAs targeting specific genes were shown in **Table S1**. A549 cell lines stably expressing STAT2-WT, STAT2-Y690F, or empty vector (EV) were generated by infecting the cells with retroviruses encoding these genes in pNL-CMV vector, as previously described (37).

### 2.5 RNA extraction and RT-qPCR

Total RNA was isolated from cultured cells using TRIzol reagent (Invitrogen, Carlsbad, CA, USA). According to the manufacturer’s method, reverse transcription of RNA (2 µg) into cDNA using a reverse transcription kit (Mei5bio, Beijing, MF949-01), followed by PCR using rTaq DNA polymerase and quantitative PCR (qPCR) using SYBR PremixEx TaqII (TaKaRa, Tokyo, Japan). GAPDH was chosen as a reference gene for internal standardization. The normalized ratio indicates that the ratio is automatically calculated by the Light Cycler system (Roche, Switzerland) using the ΔΔCT method. And the primers for RT-PCR and RT-qPCR were shown in **Table S2**.

### 2.6 Western blotting

Cell lysis was performed on collected cell samples using Beyotime (Shanghai, ST506). The protein concentration was quantified by the BCA protein assay kit (Beyotime, Shanghai, P0012). Finally, the protein samples were separated by SDS-PAGE and transferred to the NC membrane (Merck Millipore, HATF00010) for detection with an appropriate proportion of TBS diluted primary antibody.

### 2.7 Hemagglutinin and plaque assay

For hemagglutinin (HA) assay, cell culture supernatant was collected at indicated time point after virus infection, adding 25 μL PBS, and then adding the 25 μL diluted virus sample to the first well, successively diluting it from left to right. Then added 25 μL 1% chicken red blood cells to all wells. At last, we incubated the samples for 20 minutes to read the results.

For plaque assay, MDCK cells were infected with supernatant of influenza virus culture and cultured at 37°C for 1 h. The cells were then washed with PBS to remove the virus inoculants and covered with a mixed medium containing low-melting-point agarose (Promega, Madison, WI, USA) and 1 µg/mL tosylamido phenylethyl chloromethyl ketone (TPCK)-treated trypsin (Sigma-Aldrich, St. Louis, MO, USA). Plaque was visible in 2 or 3 days at 37°C, and viral titers were counted and determined.

### 2.8 Statistical analysis

All data were represented by means and standard deviations (SD) from three independent experiments. GraphPad Prism 8 (GraphPad Software, Inc.) was used for all statistical evaluations. The student’s t-test was used to analyze the statistical difference between the two groups. P < 0.05 (shown by ∗), P < 0.01 (shown by ∗∗), P < 0.001 (shown by ∗∗∗), P < 0.0001 (shown by ∗∗∗∗). NS stands for not significant.

## 3 Results

### 3.1 Phosphorylation of STAT2 can be induced by several viruses at early infection stage

**Figure 1:**
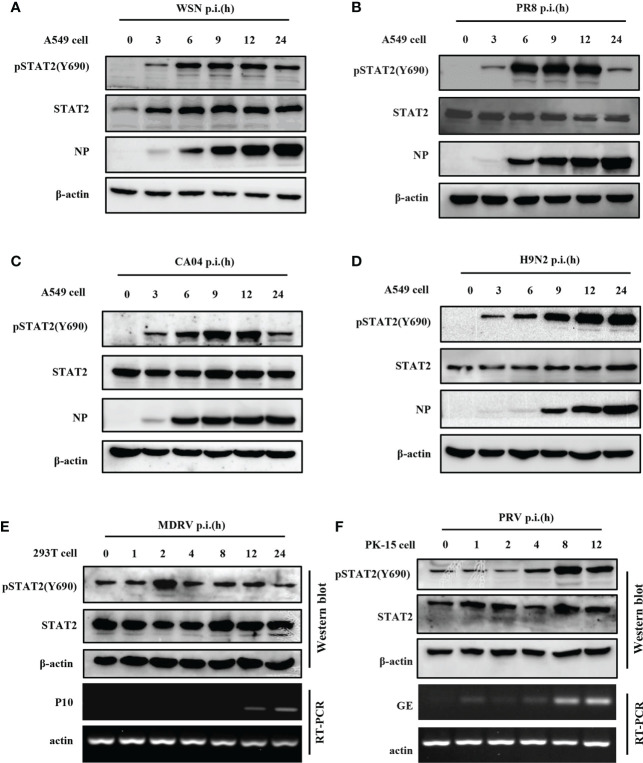
Phosphorylation of STAT2 can be induced by several viruses at early infection stage. **(A–D)** A549 cells were infected with IAV strains, including WSN **(A)**, PR8 **(B)**, CA04 **(C)**, and H9N2 **(D)** at indicated time points. Cell lysates were collected to detect the corresponding protein by Western blotting. NP, IAV nucleoprotein. Diagram shows representative data from three independent experiments. **(E, F)** 293T cells were infected with MDRV **(E)** and PK15 cells infected with PRV **(F)** at corresponding time points. Corresponding proteins were detected by Western blotting, the corresponding mRNAs were detected by RT-PCR. P10, the non-structural protein of MDRV. GE, glycoprotein E of PRV.

To investigate STAT2 Y690 phosphorylation (pSTAT2Y690) induced by viral infections, we performed a time course study in which levels of pSTAT2Y690 were examined in cells infected with various strains of IAV such as WSN, PR8, CA04, H9N2, another RNA virus, Muscovy duck reovirus (MDRV), and also DNA virus such as Pseudorabies virus (PRV). Increased pSTAT2Y690 was observed after WSN (**Figure 1A**), PR8 (**Figure 1B**), CA04 (**Figure 1C**), and H9N2 (**Figure 1D**) infection with respect to indicated hours of post infection (hpi). However, the STAT2 Y690 phosphorylation declined at the late hpi in the cells infected by various strains of IAV except infection with H9N2 (**Figures 1A–D**). Sustained activation of STAT2 even at 24 hpi following H9N2 infection may be related to the subtype or virulence of the strain. Of note, infection with several IAV strains tested (WSN, PR8, CA04, and H9N2) (**Figures 1A–D**) caused an increase in pSTAT2Y690 immediately at approximately 3 hpi. Next, we examined STAT2 phosphorylation induced by other RNA virus such as MDRV. It was found that early activation of STAT2 was also induced by infection of MDRV (**Figure 1E**). Additionally, elevated level of pSTAT2Y690 was observed in cells infected with a DNA virus PRV as early as 4 hpi (**Figure 1F**). Taken together, these data demonstrated that initial activation of STAT2 could be induced by infection of several types of viruses in various cell lines.

### 3.2 Initial activation of STAT2 is independent of type I and type III IFNs signaling at the early viral infection

**Figure 2:**
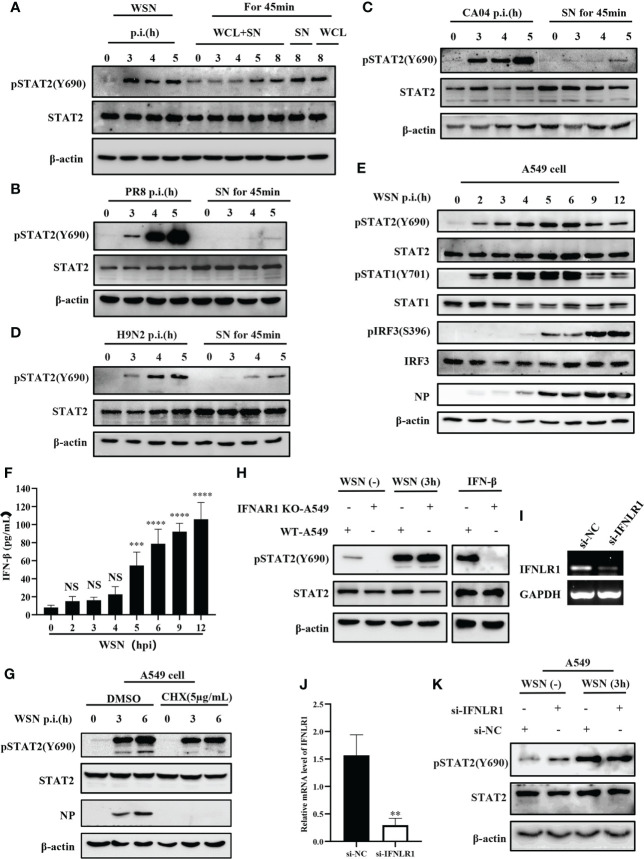
Initial activation of STAT2 is independent of type I and type III IFNs signaling at the early viral infection. **(A)** A549 cells were infected with WSN at the indicated time. Supernatant and whole cell lysate (WCL+SN) at the corresponding time point were collected to stimulate the naive cells for 45min, respectively. In the figure, SN and WCL were used as controls for the early phosphorylation of STAT2 Y690. The pSTAT2(Y690) was detected by Western blotting. **(B–D)** A549 cells were infected with different influenza strains PR8 **(B)**, CA04 **(C)**, and H9N2 **(D)**. Corresponding cell supernatant (SN) cultures were collected to stimulate the naive cells for 45 min, respectively, followed by Western blotting with indicated antibodies. **(E, F)** A549 cells were infected with WSN for indicated time points. Western blotting analysis was performed to detect the phosphorylation of STAT1, STAT2, and IRF3 **(E)**. ELISA was employed to examine the protein levels of IFN-β in the SN **(F)**. **(G)** A549 cells were treated with DMSO or cycloheximide (CHX) (5µg/mL) for 20 min in advance, then infected with WSN for indicated time. pSTAT2 was detected by Western blotting. **(H)** IFNAR1 knockout (IFNAR1-KO-A549) or wild-type A549 (WT-A549) cells were infected with WSN for 3 h or treated with IFN-β for 45 min. pSTAT2 was analyzed by Western blotting. **(I, J)** Si-IFNLR1 or control si-NC was transfected into A549 cells for 24 **(h)** RT-PCR **(I)** and RT-qPCR **(J)** were used to detect the knockdown efficiency. **(K)** Si-IFNLR1 or si-NC -based A549 cells were infected with WSN for 3 h, and the level of pSTAT2(Y690) was detected by Western blotting. Data represent the mean ± SD from three independent experiments. **p < 0.01. The above results show the representative results of three independent experiments. ***p <0.001; ****p <0.0001; ns, no significance.

It is well known that cytokines activate JAK/STAT signal transduction pathway (38). In particular, type I and type III IFNs are critical for activation of JAK/STAT signaling in innate antiviral immunity. Interestingly, previous report revealed that early activation of STAT1 is independent of cytokines (34). Therefore, we determined whether early phosphorylation of STAT2 Y690 upon viral infection was independent of these IFNs. A549 cells were infected by IAV at indicated time points and cell lysates were subjected to Western blotting for the examination of pSTAT2Y690 (**Figure 2A**). The results showed that phosphorylation of STAT2 Y690 appeared in cells at 3 hpi by WSN infection but was not caused by lysates of cells infected with the IAV (WCL) and cell culture supernatant (SN). Similarly, the STAT2 phosphorylation occurred earlier in A549 directly infected by other IAVs than that in A549 cells stimulated by virus-infected cell culture supernatant (**Figures 2B–D**). Furthermore, A549 cells were infected with WSN for different time points, and the phosphorylation of STAT1, STAT2 and IRF3 was examined. We observed that the phosphorylation of STAT2 and STAT1 occurred as early as 2 hpi and enhanced at 3 hpi. By contrast, the phosphorylation of IRF3 and expression of IFN-β was induced by the IAV infection at 5 hpi and later (**Figures 2E, F**). These results suggest that phosphorylation of STAT2 and STAT1 was likely induced before IRF3 activation and IFN production during IAV infection. Next, we employed cycloheximide (CHX) that can inhibit eukaryotic translation and eukaryotic protein synthesis. The data exhibited that increased phosphorylation of STAT2 still appeared at early infection stage (3 hpi) (**Figure 2G**). We further utilized type I IFN receptor knockout (IFNAR1 KO) cells and found that there was no difference in phosphorylation of STAT2 Y690 between IFNAR1 KO cells and WT A549 cells at 3 h post influenza virus infection, whereas IFN-β-induced pSTAT2Y690 was dramatically decreased (**Figure 2H**). Since STAT2 can also be activated by type III IFNs (16), we investigated whether type III IFNs are involved in virus-induced early phosphorylation of STAT2. For this, IFNLR1 siRNA was transfected into A549 cells for 24 h, followed by influenza virus infection, and protein and RNA samples were collected at the specified time. As compared with si-NC, the expression of IFNLR1 was significantly disrupted in si-IFNLR1 group (**Figures 2I–J**). However, Western blotting showed that silencing IFNLR1 had little effect on STAT2 activation at the early stage of influenza virus infection (**Figure 2K**). Together, these results suggest that type I and type III IFNs are likely not required for initial activation of STAT2 during the IAV infection.

### 3.3 Inhibition and silence of JAKs have little effect on IAV-induced early phosphorylation of STAT2 at Y690

**Figure 3:**
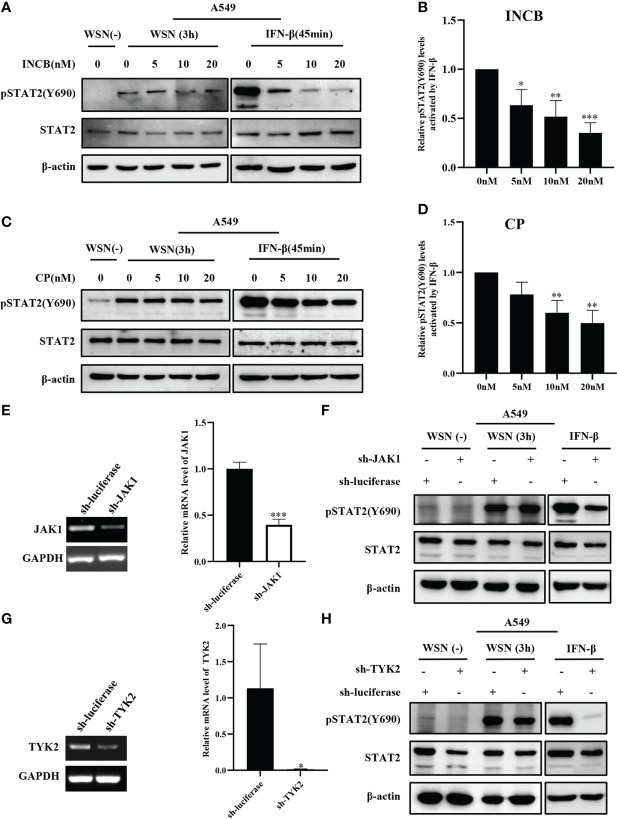
Inhibition and silence of JAKs have little effect on IAV-induced early phosphorylation of STAT2 at Y690. **(A, C)** A549 cells were pretreated with INCB018424 (INCB) **(A)** and CP-690550 (CP) **(C)** at different concentrations for 20 min, respectively, followed by WSN infection for 3 h or IFN-β (0.1 μg/mL) stimulation for 45 min. Western blotting analysis of pSTAT2 levels revealed representative blots from three independent experiments. **(B, D)** The level of pSTAT2 in **(A)**, **(C)** was quantified by densitometry and normalized to STAT2 levels. The data are shown as the mean ± SD from three independent experiments. *p < 0.05; **p < 0.01; ***p < 0.001. **(E, G)** The interference efficiencies in JAK1- **(E)** and Tyk2- **(G)** ablated and control (luciferase) A549 cells were detected by RT-PCR (Left) and RT-qPCR (Right). Error bars represent the mean ± SD from three independent experiments. *p < 0.05; **p < 0.01; ***p < 0.001. **(F, H)** A549 cells as described in **(E, G)** were infected with WSN for 3 h or stimulated with IFN-β (0.1 μg/mL) for 45 min, respectively. The expression levels of specific proteins were detected by Western blotting. The data in the figures are representative images of three repetitions.

Next, we analyzed the effect of JAKs on the early phosphorylation of STAT2 Y690. First, we used the JAK inhibitor INCB018424 (INCB) to inhibit the activation of JAK1. We found that levels of the virus-induced pSTAT2Y690 were not significantly changed by JAK inhibitor treatment at the early stage of viral infection (**Figure 3A**). On contrast, phosphorylation of STAT2 Y690 decreased significantly in INCB treated cells followed by stimulation with IFN-β (**Figures 3A–B**). Similar results were observed when cells were treated with CP-690550 (CP), another inhibitor of JAK activation, followed by IAV infection or IFN-β treatment (**Figures 3C–D**). Furthermore, we used specific shRNAs to target JAK1 in A549 cells, and knockdown efficiency was determined by RT-qPCR (**Figure 3E**). Silencing JAK1 had little effect on pSTAT2Y690 at the early stage of viral infection (**Figure 3F**), but IFN-β-stimulated pSTAT2Y690 was significantly decreased upon disruption of JAK1 expression (**Figure 3F**). Previous studies have reported that the activation of STAT2 mainly depends on JAK1 and TYK2 (39). Therefore, we further explored whether TYK2 affected the early activation of STAT2. To this end, specific TYK2 shRNAs were employed to construct TYK2 knockdown A549 cells (**Figure 3G**). Similarly, the STAT2 phosphorylation was not significantly changed at the early stage of viral infection compared with the luciferase-targeted control (**Figure 3H**). As expected, the pSTAT2 was significantly reduced in IFN-β-stimulated sh-TYK2 A549 (**Figure 3H**). The data suggest that early phosphorylation of STAT2 may be independent of JAKs during the viral infection.

### 3.4 Disruption of STAT2 phosphorylation at Tyr690 suppresses antiviral response

**Figure 4:**
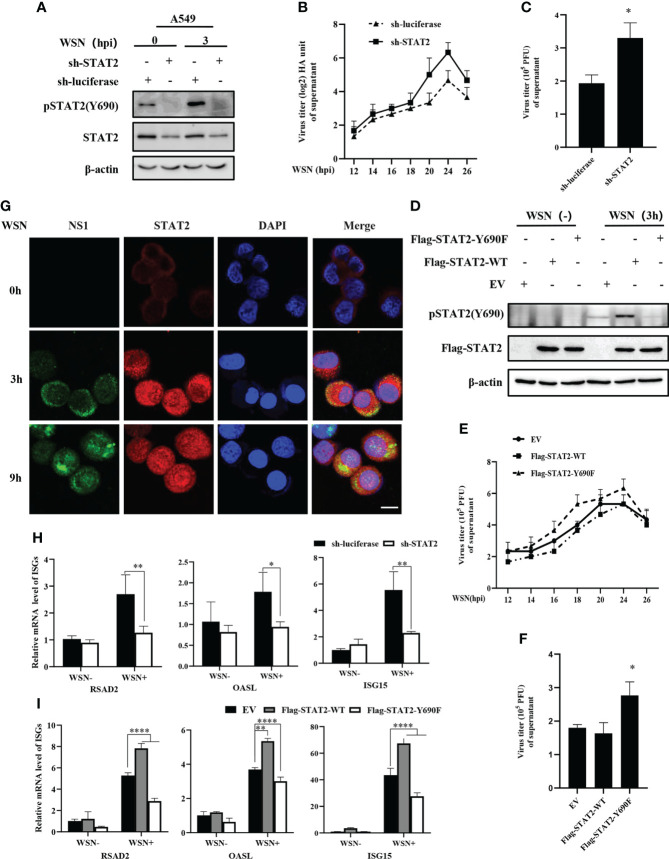
Disruption of STAT2 phosphorylation at Tyr690 promotes the IAV replication. **(A)** A549 cells stably expressing specific sh-RNA targeting STAT2 and control (luciferase) were infected with WSN virus for 3 **(H)** Cell lysates were analyzed by Western blotting with indicated antibodies. **(B, C)** STAT2- and luciferase- ablated A549 cells were infected with WSN (MOI = 1). Viral titers in supernatant of these cell cultures were examined by hemagglutinin (HA) assay at the indicated time points post infection **(B)**. Sh-RNAs knockdown A549 cells or control cells were infected with WSN as described in **(B)**. Viral titers in the supernatants of these cells were examined by plaque assay (24 h) **(C)**. The mean ± SD of three independent experiments is given. (*P < 0.05) **(D)** A549 cells stably expressing STAT2-WT, STAT2-Y690F, and empty vector (EV) control were infected with or without WSN for 3 h and examined by Western blotting with the indicated antibodies. **(E)** A549 cells stably expressing STAT2-WT, STAT2-Y690F, and empty vector (EV) control were infected with WSN for indicated time point. Hemagglutinin (HA) assay were performed to examine the viral titers in the supernatant of cell culture. **(F)** The STAT2 overexpressing A549 cells or control cells as described in **(D)** were infected with WSN. Viral titers in the supernatant of these cells culture were examined by plaque assay (24 h). Data are represented as mean ± SD. (*P < 0.05.) **(G)** A549 cells were infected with or without WSN virus for 0 h, 3 h, and 9 (h) Immunofluorescence staining was performed to detect STAT2 (red) and NS1 (green). The nuclei were stained with DAPI (blue). Scale bar, 10 μm. **(H)** RT-qPCR was performed to detect the expression of RSAD2, OASL, and ISG15 in STAT2 knockdown A549 cells with IAV infection for 3 (h) **(I)** STAT2 overexpressing or control A549 cells as described in **(D)** were infected with WSN for 3 h, and the expression of RSAD2, OASL, and ISG15 was detected by RT-qPCR. Error bars represent the mean ± SD from three independent experiments. *p < 0.05; **p <0.01; ****p <0.0001.

The tyrosine residue Tyr690 of STAT2 is directly adjacent to the domain SH2D and plays an important role in its interactions with other STAT proteins, especially STAT1 (40). In a typical IFN-I-induced signal transduction pathway, ISGF3 complex is formed by the interaction between STAT1 at Tyr701 and STAT2 at Tyr690 (17), which is crucial for antiviral immunity. Since STAT2 Y690 could be phosphorylated at the early stage of viral infection, we asked whether the phosphorylation of STAT2 had any effect on IAV replication. First, specific shRNAs were employed to target STAT2 in A549 cells (**Figure 4A** and **Figures S1A–S1B**). Supportively, STAT2 knockdown using sh-STAT2 significantly diminished IAV-induced activation of STAT2 at Tyr690 at 3 hpi (**Figure 4A**), and resulted in enhanced virus replication and higher viral titer as compared to the control (**Figures 4B–C**), suggesting that STAT2 is essential for establishment of antiviral response. To verify this observation, STAT2 Y690F, a dominant negative mutation of STAT2 (41) was generated (**Figure 4D**). We found that overexpression of STAT2 Y690F significantly enhanced the IAV replication as compared to that in cells expressing STAT2 WT or EV control (**Figures 4E, F**).

Then, we examined the translocation of STAT2 into the nucleus after WSN infection. The accumulation of STAT2 and pSTAT2Y690 in the nucleus was observed as early as 3 hpi (**Figure 4G**, **Figure S1C**). To further address the relevance of early pSTAT2Y690 translocation into the nucleus with the activation of STAT2, we evaluated the expression of ISGs in STAT2 knockdown cells, and in the cells expressing WT or the dominant negative mutant of STAT2 (STAT2 Y690F) at the early time point post WSN infection. As shown in **Figures 4H, I** depletion of STAT2, or disruption of the STAT2 phosphorylation at Tyr690 led to a significant decrease in the expression of ISGs such as RSAD2, ISG15, and OASL at 3 hpi. These results suggest that STAT2 phosphorylation at Tyr690 accompanied by the initial activation of STAT2, contribute to the antiviral ISG response during early IAV infection. Overall, it was revealed that activation of STAT2 at Tyr690 played a critical role in host innate immune response to IAV infection.

### 3.5 Early activation of STAT2 is mainly regulated by RIG-I/MAVS signaling

**Figure 5:**
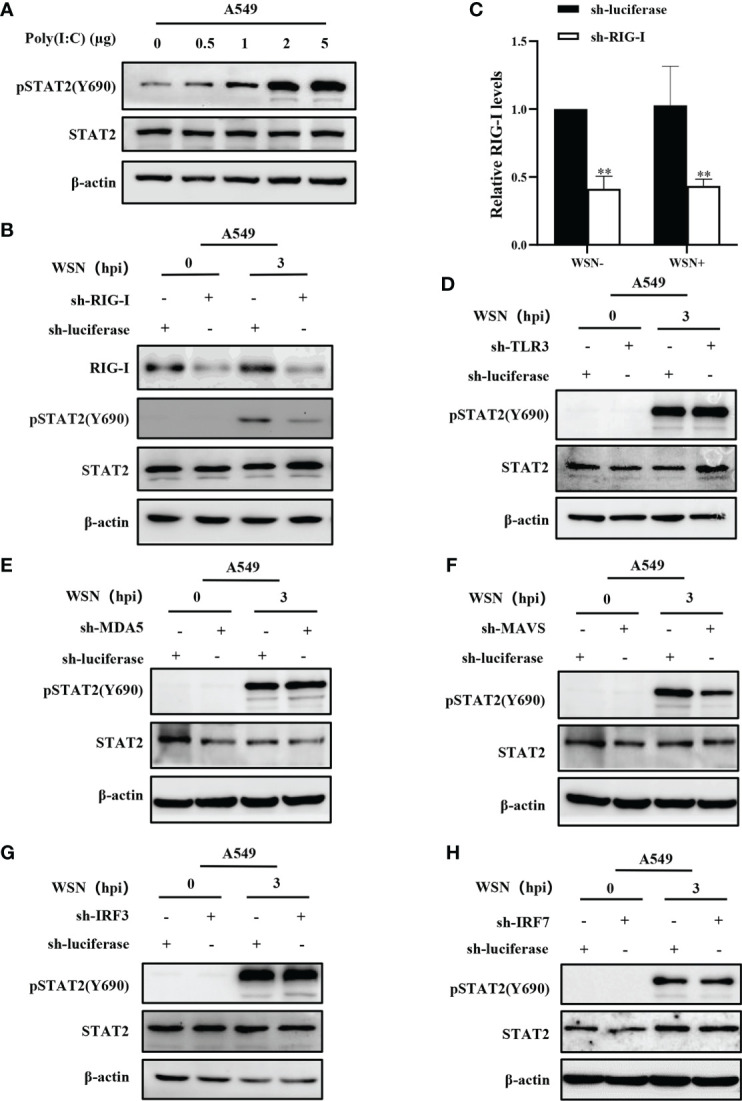
Early activation of STAT2 is mainly regulated by RIG-I/MAVS signaling. **(A)** A549 cells were transfected with different concentrations of Poly(I:C) for 6 h, and pSTAT2 were analyzed by Western blotting. **(B ,C)** Western blotting was performed to evaluate the indicated proteins in WSN-infected RIG-I or control (luciferase) knockdown A549 cells **(B)**. The RIG-I levels were quantitated by densitometry as described in B **(C)**. Error bars represent the mean ± SD from three independent experiments. **p < 0.01. **(D–H)** The sh-RNA based A549 cell lines stably expressing TLR3 **(D)**, MDA5 **(E)**, MAVS **(F)**, IRF3 **(G)**, IRF7 **(H)**, and control (luciferase) were infected with WSN (MOI =1) for 3 h Cell lysates were collected and pSTAT2 levels were detected by Western blotting **(D–H)**. The results shown in the figure are representative results of three independent experiments.

The innate immune system triggers an antiviral response upon sensing various viral components, including double-stranded RNA by PRRs. Cytoplasmic helicase proteins RIG-I and MDA5 are main PPRs in sensing particular RNA viruses such as IAV (42). Although IAV genomic RNA panhandle structure has been shown to be recognized by RIG-I, TLR3, TLR7, and TLR8 (8, 43), it remains to be determined whether early phosphorylation of STAT2 is activated by the PRR-dependent signaling. To determine whether STAT2 Y690 can also be activated by dsRNA in host cells, we transfected A549 with different concentrations of poly (I:C), the dsRNA analogues for 6 h. Increased phosphorylation of STAT2 at Y690 was observed with respect to the increasing concentration of poly (I:C) (**Figure 5A**). Next, RIG-I, MDA5, or TLR3 knockdown A549 cell lines were generated, respectively (**Figures S2A–S2D**). The result showed that silencing RIG-I, but not MDA5 or TLR3, significantly reduced early pSTAT2Y690 levels (**Figures 5B–E**), suggesting that RIG-I was involved in IAV-indued early activation of STAT2. To study the specific pathway through which early activation of STAT2 occurred, we generated MAVS, IRF3 or IRF7 knockdown A549 cells followed by IAV infection (**Figures S2E–** **G**). The results showed that early STAT2 activation was significantly reduced in sh-MAVS cells (**Figure 5F**). However, there was no significant change of pSTAT2Y690 in sh-IRF3 and sh-IRF7 cell lines after the IAV infection (**Figures 5G–H**). In addition, we also found that the early phosphorylation of STAT2 was not affected when P65 of NF-κB was inhibited by BAY 11-7082 (**Figure S2H**). Therefore, the data suggest that the phosphorylation of STAT2 Y690 at the early stage of viral infection was mainly regulated through the RIG-I/MAVS signaling.

### 3.6 Several kinases are involved in early activation of STAT2 induced by IAV infection

**Figure 6:**
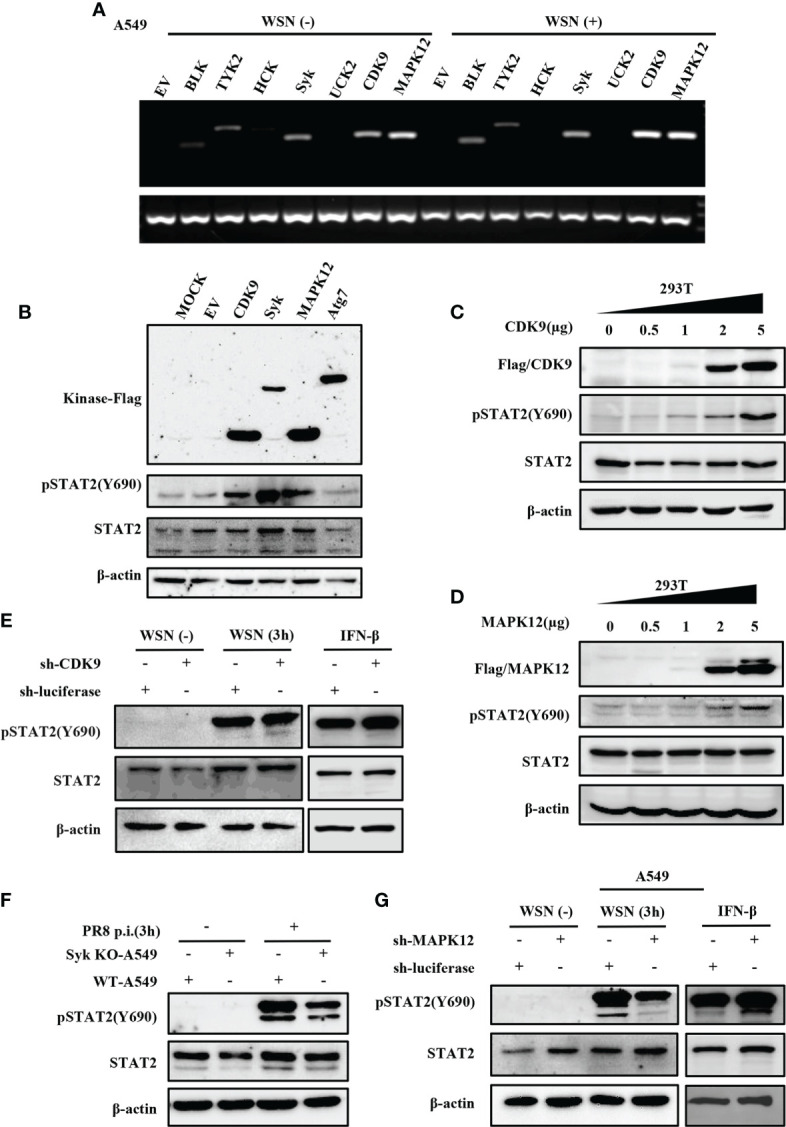
Several kinases are involved in early activation of STAT2 induced by IAV infection. **(A)** A549 cells were infected with or without WSN for 3 h, and the expression of various kinases was detected by RT-PCR. **(B)** 293T cell lines were transfected with different kinase plasmids for 24 h (MOCK as blank control, EV and Atg7 as negative control). Western blotting was performed to evaluate the pSTAT2 levels. **(C, D)** 293T cells were transfected with increasing amounts of MAPK12 **(D)** and CDK9 **(C)** -expressing plasmids as indicated or empty vector as control for 24 (h) Western blotting was performed with the indicated antibodies. Plotted are the representative data of three repeated experiments. **(E, G)** Sh-RNA-based A549 cells lines stably expressing CDK9 **(E)**, MAPK12 **(G)**, or control (luciferase) were infected with WSN with (MOI =1) for 3 h or stimulated by IFN-β (0.1 μg/mL) for 45 min, and the corresponding protein levels were detected by Western blotting. **(F)** Syk knockout A549 cells (Syk KO-A549) or control cells (WT-A549) were infected with PR8 (MOI =1) for 3 h and then protein samples were collected. The pSTAT2 was detected by Western blotting. The above data are representative results of three independent experiments.

Since the virus-induced early activation of STAT2 is not regulated by JAKs, we identified which kinase(s) might regulate this process. Thus, we employed a kinase library and transfected the kinases into 293T cells to screen for kinases that could mediate the efficient phosphorylation of STAT2 Y690 (Data not shown). Then, RT-PCR was used to detect the kinases that could be expressed in A549 cells infected with or without WSN (**Figure 6A**). Syk, CDK9, and MAPK12 were preliminarily selected based on their relatively higher expression levels in A549 cells and ability to phosphorylate STAT2 in 293T cells (**Figures 6A, B** and **Figure S** **3A**). Furthermore, transfection of plasmids expressing these kinases in increasing concentrations caused an increased activation of STAT2 with respect to plasmid concentrations (**Figures 6C–D** and **Figure S3B**). To further investigate kinases involved in STAT2 activation at the early stage of viral infection, we constructed CDK9 or MAPK12 knockdown A549 cells using specific shRNAs, respectively (**Figures S3C–S3E**). The Syk knockout A549 cells were produced by our laboratory (44). The data showed that deleting Syk and silencing MAPK12 but not CDK9 caused a diminished early activation of STAT2 (**Figures 6E–G**), indicating that MAPK12 and Syk were key kinases for early activation of STAT2 induced by IAV infection.

### 3.7 MAPK12 affects the expression of some ISGs during influenza virus infection

**Figure 7:**
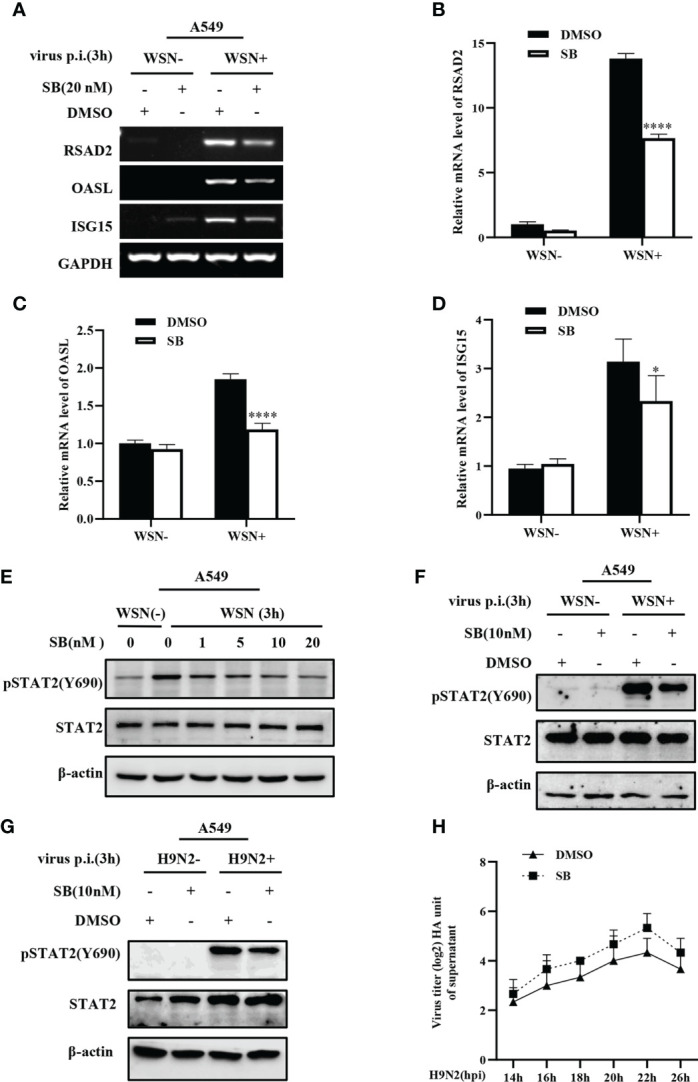
MAPK12 affects the expression of some ISGs during influenza virus infection. **(A–D)** SB203580 (SB) pretreated- A549 cells were infected with WSN for 3 h, and the expression of antiviral genes was detected by RT-PCR **(A)** and RT-qPCR **(B-D)**. Error bars represent the mean ± SD from three independent experiments. *p < 0.05; ****p <0.0001. **(E)** A549 cells were pretreated with SB203580 (SB) at indicated concentrations for 20 min, then infected with WSN for 3 h, and pSTAT2 level was detected by Western blotting. Shown are representative data from three independent experiments. **(F, G)** SB203580 (SB) pretreated-A459 cells were infected with WSN **(F)** or H9N2 **(G)** for 3 h, and the level of pSTAT2 was detected by Western blotting. **(H)** SB203580 (SB) pretreated-A459 cells or control (DMSO) cell lines were infected with H9N2. Viral titers in supernatant of these cell culture were examined by hemagglutinin (HA) assay at indicated time point.

MAPK12 is an important kinase involved in various signal transduction pathway. Considering the key role of MAPK12 in innate immune response, we further asked whether MAPK12 was involved in the regulation of antiviral genes during early viral infection. For this, we determined the expression of antiviral genes in cells treated with SB203580, an inhibitor of MAPK12 and control cells. It was found that inhibition of MAPK12 resulted in decreased expression of antiviral genes, such as RSAD2, OASL, and ISG15 (**Figure 7A**). The above results were further verified by RT-qPCR (**Figures 7B–D**). This indicated that MAPK12 positively regulates the expression of early antiviral genes. Next, we examined the early activation of STAT2 induced by IAV infection in A549 cells by treating with increasing concentration of SB203580. It was observed that the decreased phosphorylation level of STAT2 was correlated with the concentration of MAPK12 inhibitor (**Figures 7E–F**). Furthermore, we found that H9N2 induced pSTAT2Y690 was also decreased after MAPK12 inhibition (**Figure 7G**). It has been reported that RIG-I could activate p38 MAPK during SeV or Japanese encephalitis virus infection (45, 46). Our results showed that forced expression of MAVS led to a significant increase in the phosphorylation of STAT2 (**Figure S4**), implying that the RIG-I/MAVS signaling may mediate the activation of MAPK12 (p38γ), thereby enhancing early STAT2 phosphorylation. In addition, hemagglutinin assay showed that inhibition of MAPK12 remarkably promoted the replication of H9N2 virus (**Figure 7H**), suggesting that MAPK12 played an important role in host antiviral response. Taken together, MAPK12-mediated phosphorylation of STAT2 Y690 is critical for innate antiviral immunity at the early stage of influenza virus infection.

## 4 Discussion

Our previous studies have shown that STAT1 can be activated through RIG-I/MAVS/Syk pathway at the early stage of viral infection, which is independent of cytokines and JAK signaling pathways (34). Similar to other members of the STAT family, STAT2 plays an important role in host defense against viral infections (17). In the JAK-STAT pathway mediated by type I IFN, phosphorylation of STAT1 and STAT2 leads to heterodimerization and interaction with IRF9, resulting in the formation of ISGF3 which then translocates into the nucleus and induces the transcription of ISGs (21, 47). STAT2 plays a key role in mediating antiviral immunity, as STAT2^-/-^ mice exhibit increased susceptibility to viral infection and impaired response to type I IFN (48). Similar to STAT1^-/-^ or IFNAR1^-/-^ mice (49, 50), STAT2^-/-^ mice display excessive inflammation, viral burden, and increased morbidity after infection with influenza virus (51). In addition, STAT2^-/-^ hamsters have significantly elevated pulmonary viral titers after SARS-COV-2 infection (52). These observations indicate that disruption of STAT2 expression *in vivo* leads to a broad spectrum of viral susceptibility and higher mortality in animals. Besides, it has been shown that STAT2 positively regulates the induction of IL-6 in cooperation with the NF-κB pathway, and participates in the negative feedback of type I IFN signaling (53, 54). These results suggest that STAT2 plays a multitude of roles in regulating innate immunity and virus-host interactions.

Notably, it has been reported that IFN-like transcriptomes occur in the absence of IFN signaling in response to viral infection (55), which provides evidence for ISGs production in the absence of IFN during the early phase of viral infection. In this study, we observed that early phosphorylation of STAT2 at Y690 is activated by different IAV strain including WSN, PR8, CA04, and H9N2. Interestingly, initial activation of STAT2 was also detected upon infections with several other viruses such as dsRNA virus MDRV, and DNA virus PRV. Moreover, our results demonstrated that early activation of STAT2 was independent of JAKs, IFNAR1, and IFNLR1 following IAV infection, indicating that the initial activation of STAT2 is independent of the cytokine-mediated JAK signaling. This prompted us to probe signaling pathways that contribute to the early activation of STAT2. We found that depletion of RIG-I, but not TLR3 or MDA5, significantly dampened the initial activation of STAT2. As the primary mediator of RIG-I signaling (56), MAVS is required for the transduction of RIG-I signaling and consequent initiation of IFN responses (57, 58). Our results showed that silencing MAVS remarkably impaired the initial activation of STAT2, indicating that RIG-I/MAVS pathway mediates the early activation of STAT2 during IAV infection. NF-κB, IRF3, and IRF7, are the key transcription factors to induce the expression of type I and type III IFNs (59). By contrast, our results showed that silencing NF-κB, IRF3 or IRF7, had no significant effect on STAT2 phosphorylation at the early stage of influenza virus infection, supporting that cytokines are not required for the initial activation of STAT2 following IAV infection. Furthermore, IAV-induced phosphorylation of STAT2 Y690 plays an important role in antiviral response, as disruption of STAT2 expression, or forced expression of the dominant negative mutant of STAT2 (STAT2 Y690F) significantly decreased the expressions of several key ISGs such as RSAD2, ISG15, and OASL, thereby facilitating the replication of IAV.

In an attempt to identify potential kinase(s) that regulate the phosphorylation of STAT2 at Y690 at the early stage of viral infection, we found that Syk and MAPK12 may be involved in the early activation of STAT2 upon IAV infection, as depletion of Syk or MAPK12 significantly decreased STAT2 activation at the early stage of viral infection. MAPK12 (p38γ) is one of the p38 MAPKs which play an important role in the signaling cascades critical for innate immune responses (60–62). MAPK12-/- mice produced lower levels of cytokines such as IL-1β (63) and TNF-α upon LPS stimulation (62), thereby exhibiting lower symptom severity and joint damage compared with wild type mice. We then evaluated the functional involvement of MAPK12 in the host antiviral immunity in response to IAV infection. Our results showed that inhibition of MAPK12 dramatically impaired the expression of ISGs such as RSAD2, ISG15, and OASL, thereby promoting the replication of IAV. These results indicate that MAPK12 may be a kinase that mediates STAT2 activation at the early stage of influenza virus infection. Since MAPK12 is a serine/threonine kinase (64), it is possible that MAPK12 might modulate the activation of an unidentified tyrosine kinase that is responsible for the phosphorylation of STAT2 at Y690 during the early stage of viral infection. In addition, previous studies have shown that RIG-I and TLR3 can mediate the activation of p38 MAPKs during SeV or Japanese encephalitis virus infection (45, 46). Further studies are needed to determine the involvement of RIG-I/MAVS signaling in the activation of MAPK12 during IAV infection. In addition, precise mechanisms underlying the regulation of MAPK12 activation and phosphorylation of STAT2 by MAPK12 during viral infection deserve further investigation.

In summary, our results revealed an important role of STAT2 phosphorylation at the early stage of viral infection. Moreover, we demonstrated that the initial phosphorylation of STAT2 is dependent on RIG-I/MAVS/MAPK12 pathway. These findings proposed a novel mechanism by which STAT2 repressed viral infection, and provide new insights into interactions between viruses and host innate immunity.

## Data availability statement

The original contributions presented in the study are included in the article/**Supplementary Material**. Further inquiries can be directed to the corresponding authors.

## Author contributions

SSL, J-LC, and XL conceived and designed the experiment. XL, SYL, and WZ performed the experiments. KRR performed data analysis. SW and XC processed and typeset the figures. XL, SSL, J-LC, and GG contributed to the writing of the manuscript. All authors contributed to the article and approved the submitted version.

## Funding

This work was supported by National Key Research and Development Program of China (2021YFD1800205) and National Natural Science Foundation of China (U1805231, 32030110 and 32102688).

## Acknowledgments

We thank members of J-LC’s lab in College of Animal Sciences, Fujian Agriculture and Forestry University for technical assistance and useful discussions.

## Conflict of interests

The authors declare that the research was conducted in the absence of any commercial or financial relationships that could be construed as a potential conflict of interest.

## Publisher’s note

All claims expressed in this article are solely those of the authors and do not necessarily represent those of their affiliated organizations, or those of the publisher, the editors and the reviewers. Any product that may be evaluated in this article, or claim that may be made by its manufacturer, is not guaranteed or endorsed by the publisher.
