# Supplementary material for: Initial activation of STAT2 induced by IAV infection is critical for innate antiviral immunity
Source: Front Immunol. 2022 Sep 5;13:960544. doi: 10.3389/fimmu.2022.960544 (PMC9486978; doi:10.3389/fimmu.2022.960544)
Supplement: Supplementary file 1 [file DataSheet_1.pdf]

Fig. S1

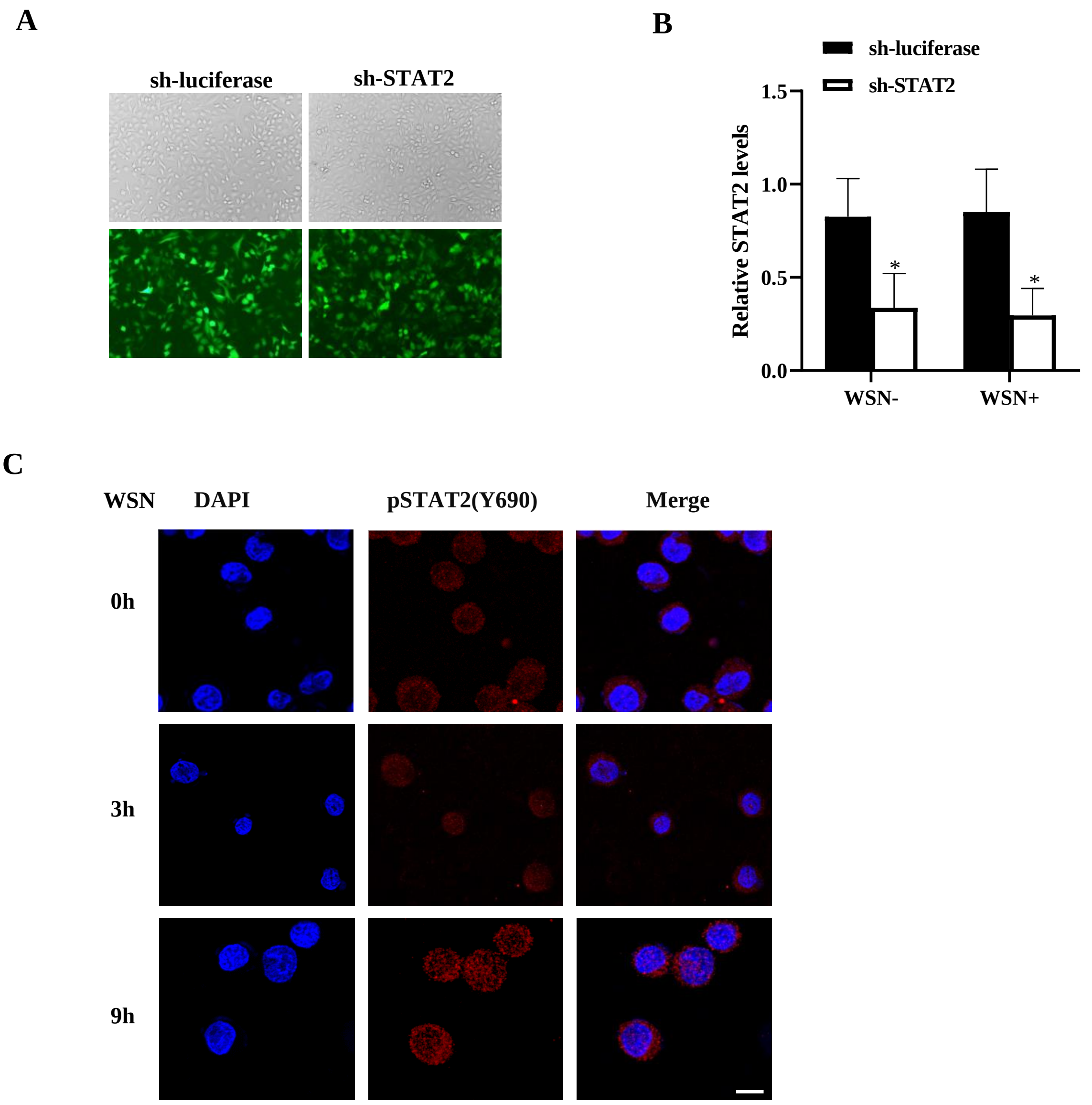

Fig. S1 Construction of sh-STAT2 based A549 cells. (A) Optical and corresponding fluorescence images of A549 cells stably expressing specific sh-RNA targeting STAT2 and control (luciferase). **(B)** The level of STAT2 in (Fig. 4A) was quantified by densitometry and normalized to control β-actin levels. Error bars represent the mean±SD from three independent experiments. *$p < 0.05$. **(C)** A549 cells were infected with or without WSN virus for 0 h, 3 h, and 9 h. Immunofluorescence staining was performed to detect the translocation of pSTAT2(Y690) (red). The nuclei were stained with DAPI (blue). Scale bar, 10 μm.

Fig. S2

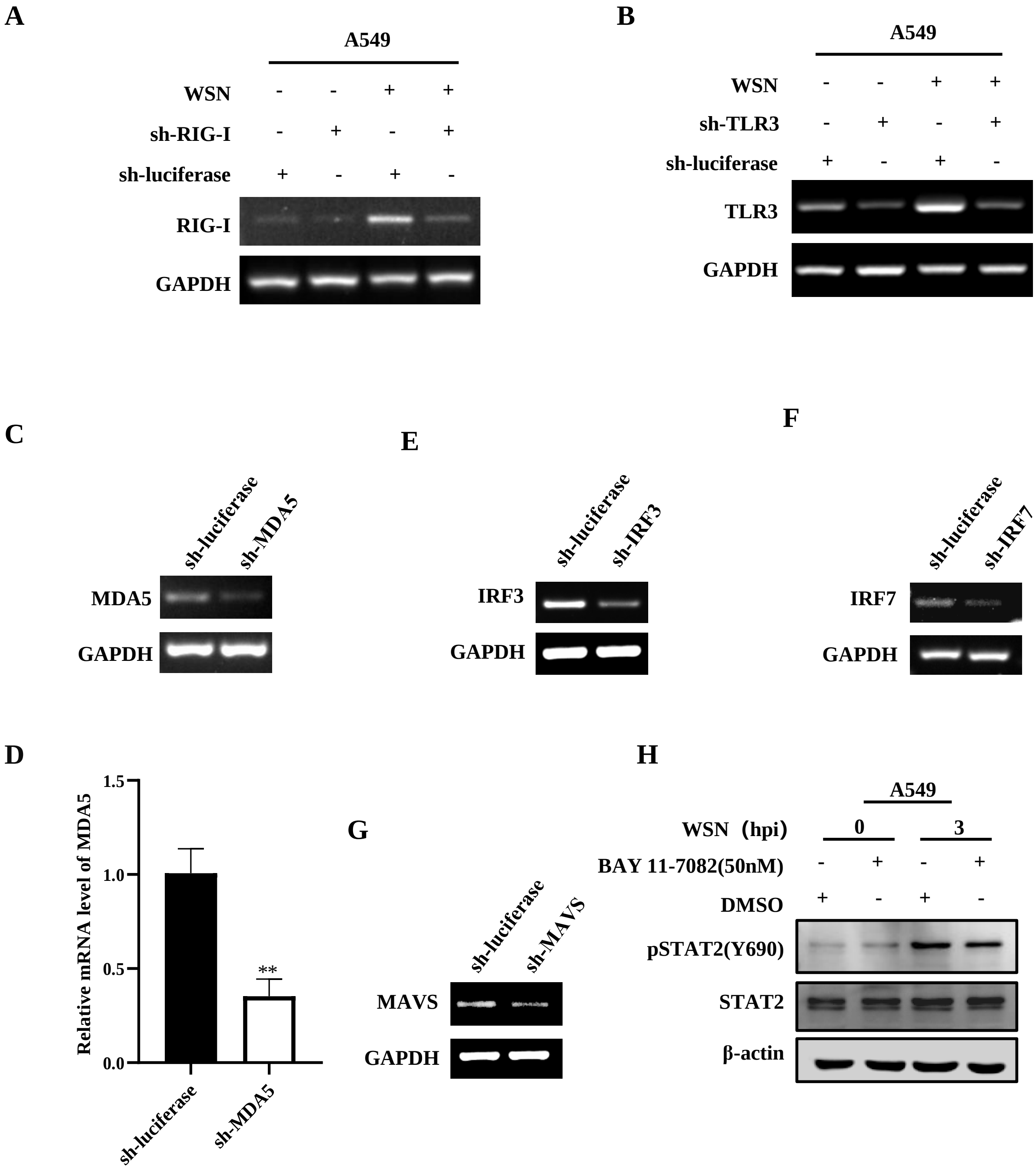

Fig.S2 Early activation of STAT2 is mainly regulated by RIG-I/MAVS signaling. (A-G) A549 cells were steadily expressed by RIG-I (A), TLR3 (B), MDA5 (C), MAVS (G), IRF3 (E), IRF7 (F), and control (luciferase) with specific sh-RNAs, and the knockdown efficiencies were detected by RT-PCR (A-C, E, F, and G) and RT-qPCR (D). (H) BAY 11-7082 pretreated-A459 cells were infected with WSN for 3 h and the level of pSTAT2 was detected by Western blotting.

Fig. S3

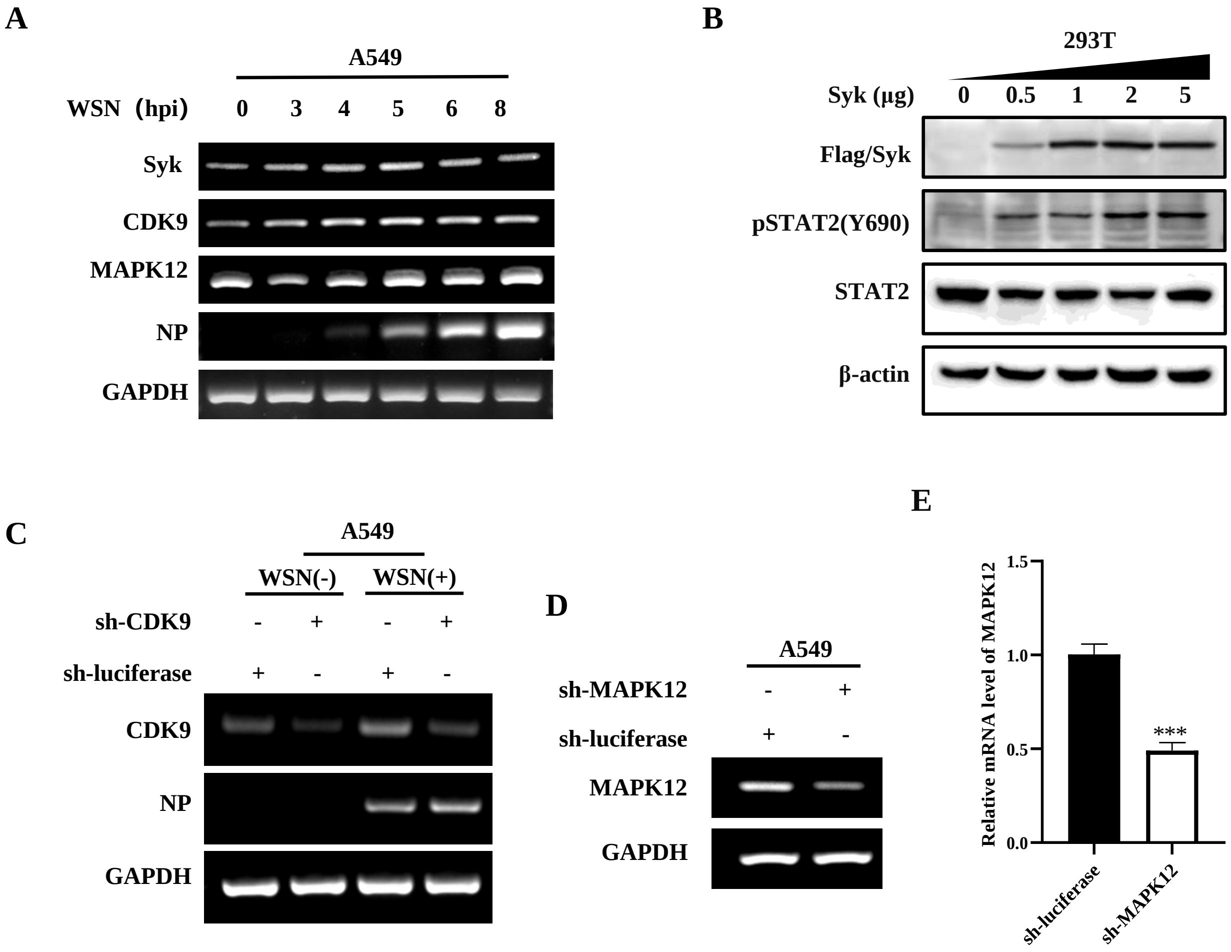

Fig.S3 Several kinases are involved in early activation of STAT2 induced by IAV infection. (A) RT-PCR was performed to detect indicated genes in WSN-infected A549 cells. (B) 293T cells were transfected with increasing amounts of Syk-expressing plasmids as indicated for 24 h, pSTAT2 was detected by Western blotting. (C-E) The knockdown efficiencies of CDK9 (C), MAPK12 (D), and control (luciferase) -ablated A549 cells were detected by RT-PCR (C and D) and RT-qPCR (E). Error bars represent the mean±SD from three independent experiments. ***p < 0.001.

Fig. S4

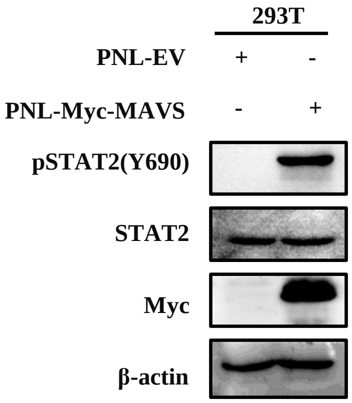

Fig. S4 Over-expression of MAVS induces STAT2 phosphorylation. 293T cells were transfected with myc-MAVS plasmid for 24 h, and Western blotting were performed to detect the phosphorylation of STAT2.
