# Supplementary material for: Initial activation of STAT2 induced by IAV infection is critical for innate antiviral immunity
Source: Front Immunol. 2022 Sep 5;13:960544. doi: 10.3389/fimmu.2022.960544 (PMC9486978; doi:10.3389/fimmu.2022.960544)
Supplement: Supplementary file 2 [file Table_1.pdf]

Table S1 Nucleotide sequences of Oligos used throughout this study		
**Primer Name**	**Primer Sequence (5'-3')**	**Source**
human shRNA-STAT2	GCCCAATTGGTGAGGACAATT	This study
human shRNA-RIG-I	GCAGAGAAATTGGTGGAATGC	This study
human shRNA-IRF7	GGAACATTGTCTGGGTCAGAT	This study
human shRNA-MAVS	GCCCAGAGGAGAATGAGTATA	This study
human shRNA-JAK1	GCATGGAACCAACGACAATGA	This study
human shRNA-TYK2	GCAGATGGTCATGGTCAAATA	This study
human shRNA-CDK9	GGTGATGCAGATGCTGCTTAA	This study
human shRNA-MAPK12	GGGCAAGCTCATGAAACATGA	This study
