# Supplementary material for: Initial activation of STAT2 induced by IAV infection is critical for innate antiviral immunity
Source: Front Immunol. 2022 Sep 5;13:960544. doi: 10.3389/fimmu.2022.960544 (PMC9486978; doi:10.3389/fimmu.2022.960544)
Supplement: Supplementary file 3 [file Table_2.pdf]

Table S2 Nucleotide sequences of primers used in this study	
**Primer Name**	**Primer Sequence (5'-3')**
qRT-human IFNLR1 F	CCAGAACTTCAGCGTGTACC
qRT-human IFNLR1 R	ACATAGCAGCTCCTTGGTTC
RT-human RIG-I F	GAGAAATTGGTGGAATGCCT
RT-human RIG-I R	ACAACTTTCCCCTTTTGTCC
RT-human TLR3 F	TGGGCCTTAATGAAATTGGG
RT-human TLR3 R	AGTTTCTCAAGACCCTCCAA
RT-human MDA5 F	CTGCTGAGCACCTTGGAGAA
RT-human MDA5 R	GAGTGGGCTGAAGGAGGTTC
RT-human IRF3 F	TCCCCAAGGACAAGGAAGGA
RT-human IRF3 R	TAGGCCTTGTACTGGTCGGA
RT-human IRF7 F	ATACCATCTACCTGGGCTTC
RT-human IRF7 R	ATGTCGTCATAGAGGCTGTT
RT-human MAVS F	GCTGTGAGCTAGTTGATCTC
RT-human MAVS R	TTCTCTCTGCAGCTGTTGTA
RT-human JAK1 F	GAGAGGCACGAGAACACACA
RT-human JAK1 R	AGATCCAGAGGACCCCCTTC
qRT-human JAK1 F	GGTATGACCCCGAAGACAAT
qRT-human JAK1 R	TACCATTTCCTCCGTCTTCT
RT-human TYK2 F	GAAAGCCCTCAAGGCAGACT
RT-human TYK2 R	TGCGTCAGCAGCTCATACAG
qRT-human TYK2 F	CACTCCTCCTTGCTTCAATC
qRT-human TYK2 R	TCTGGGGATCTCTAGGATGT
qRT-human MAPK12 F	GCTGAGGTATATCCACGCTG
qRT-human MAPK12 R	CCAGTCATCTCACTGTCTGC
qRT-human CDK9 F	CAAGATCCTCCAGCTCCTAA
qRT-human CDK9 R	TTGACTAAGACGTTGCTCAG
RT-human RSAD2 F	TCTGAGCTCTCTGTGGAGGAG
RT-human RSAD2 R	CTGTGTGGAAACAGAAGCCG
RT-human OASL F	GGGCCTTCTCTTCCCAACTC
RT-human OASL R	ATCCAGGATGATGGGCCTCT
RT-human ISG15 F	GACATCTGGAAGGAGACAGGT
RT-human ISG15 R	CCTTGAGGGTCTCCCTGCTA
qRT-human RSAD2 F	CTGCTAGCTACCAAGAGGAG
qRT-human RSAD2 R	GAAGTGATAGTTGACGCTGG
qRT-human OASL F	TTCAGCGAGCTGCAGAGAAA
qRT-human OASL R	AAGAGCATAGAGAGGGGGCA
qRT-human ISG15 F	AGATCACCCAGAAGATCGG
qRT-human ISG15 R	GTTATTCCTCACCAGGATGC
